# Caregiving, Employment and Social Isolation: Challenges for Rural Carers in Australia

**DOI:** 10.3390/ijerph15102267

**Published:** 2018-10-16

**Authors:** Rafat Hussain, Stuart Wark, Peta Ryan

**Affiliations:** 1 ANU Medical School & Research School of Population Health, Australian National University, Canberra 2600, Australia; 2 School of Rural Medicine, University of New England, Armidale 2350, Australia; swark5@une.edu.au (S.W.); pryan9@une.edu.au (P.R.)

**Keywords:** care givers, chronic illness, community care, disability, rural, remote

## Abstract

Australia has one of the world’s highest life expectancy rates, and there is a rapidly growing need for informal caregivers to support individuals who are ageing, have chronic illness or a lifelong disability. These informal carers themselves face numerous physical and psychological stressors in attempting to balance the provision of care with their personal life, their work commitments and family responsibilities. However, little is known about the specific challenges facing rural carers and the barriers that limit their capacity to provide ongoing support. A cross-sectional survey composed of open-ended responses and demographic/socioeconomic measures used routinely by the Australian Bureau of Statistics (ABS) and the Australian Institute of Health & Welfare (AIHW) was used with a cohort of 225 rurally-based carers within New South Wales, Australia. Demographic questions specified the respondents’ age, gender, employment, caregiving status, condition of and relationship to the care recipient, postcode, residency status, and distance and frequency travelled to provide care. Open-ended comments sections were provided to allow participants to describe any issues and problems associated with caregiving including employment, travel, residency, carer support groups and any other general information. The results show that most rural carers were middle-aged women supporting a spouse or a child. Unpredictability associated with providing care exacerbated demands on carers’ time, with many reporting significant employment consequences associated with inflexibility and limited job options in rural locations. Specific issues associated with travel requirements to assist with care were reported, as were the impacts of care provision on the respondents’ own personal health. The majority of carers were aware of the social supports available in their local rural community, but did not access them, leaving the carers vulnerable to marginalisation. Problems associated with employment were noted as resulting in financial pressures and associated personal stress and anxiety for the caregivers. While this issue is not necessarily limited to rural areas, it would appear that the lack of opportunity and flexibility evident in rural areas would exacerbate this problem for non-metropolitan residents. The participants also identified specific barriers to the provision of care in rural areas, including the significant impact of travel. Access to support services, such as carer groups, were rarely accessed due to a mix of factors including inaccessibility, poor timing and a lack of anonymity. Financially, there was considerable evidence of hardship, and there is an urgent need for a comprehensive review of government and community-based support to better meet the needs of rural carers.

## 1. Introduction

### 1.1. Background

With advances in both medical knowledge and the provision of specialist care, individuals around the world are not just living longer, but now also living longer with complex health issues [1]. There is an increasing need for informal care-giving, which is partially related to the recognised ageing of the general population [2], but the provision of lifelong family-based care is now also commonplace in situations where individuals born with disabilities or chronic health problems are surviving childhood and living into chronological middle and old age [3]. Previous models of health and personal care for people with severe disability, which often focused on formal institutionalisation, have been increasingly replaced with community-based support options supplementing informal family care [4].

Ongoing changes in family structure, such as the emergence of smaller and geographically diverse household units [5], limit the number of potential individuals to share family caregiving responsibilities. This has led to instances where the carer role has to be solely performed by a spouse or parent who may also be experiencing their own employment, health or ageing-related issues. This problem then further compounds the challenging circumstances facing the individual, their carer and other family members [6,7,8]. Given in many instances there is a sole carer, family caregiving responsibilities can adversely affect capacity for employment and can result in premature retirement from work [9]. In an analysis of the National Long-Term Care Survey in the USA, Kim et al. [10] reported that the intensity and duration of caregiving demands, combined with limited available resources, make it hard for caregivers to undertake full-time work. Thus, a vicious cycle may develop for caregivers with either complete breaks in employment or low-paid casual jobs, which in turn not only cause financial hardship in the short-term for the family but may reduce the carer’s longer-term career prospects and retirement income [11,12]. In a recent qualitative study in New Zealand, Horrell and colleagues report that carers’ employment choices are constrained and involve trading-off work and social options when faced with the need for providing care to a close family member [13].

In Australia, findings from the latest round of the Survey of Disability, Ageing and Carers (SDAC) conducted in 2015 indicate that there were almost 2.7 million Australian carers (11.6%), with 856,100 people (3.7% of total population and 31.7% of carers) aged 15 years and over who identified as primary carers. These patterns are consistent with those reported in the 2009 and 2012 rounds of SDAC [14]. These figures are similar to those reported in other countries, such as in the United States of America where one large epidemiological study found the prevalence of carers was around 12% [15]. However, another study reported a much higher rate, with 29% of the population providing care to someone who is aged, ill, or disabled [16]. In Australia, the majority of primary carers (79.3%) resided in the same household as the care-recipient. Of the primary carers not living with care recipient, over two-thirds (67.0% or around 119,400) were caring for a parent [14].

This increased demand for informal care is occurring concurrently with greater financial pressures and efficiency drivers being placed onto Australian health systems [17], thus limiting options for formal care. This has resulted in a significant rise in the need for informal caregivers since the year 2000 in Australia. The number of people receiving the Carers Payment increased by 145% between 1999 and 2007, with associated expenditure increased by 283% [18], and this trend has continued with Carers Payment recipient numbers increasing by 9.6% per annum from 2006 to 2016 [19].

Although patterns of caregiving are shifting in line with changing family structure and norms, there is a strong gendered dimension to caregiving. Australian government data from the past decade reports that approximately 70% of carers in Australia are women, with the largest cohort aged 45–54 [19]. Approximately three quarters of people defined as young carers (under 25 years) are female, and 98% of young carers with a child are women [20]. Data from the latest SDAC [14] shows that the overall proportion of men providing care has increased, but only marginally, with females still comprising 68.1% of primary carers. Furthermore, among the 55–64 year age group the proportion of female primary carers was almost double that of male carers (13,450 and 70,800 respectively).

The provision of care in rural areas presents novel difficulties that may not be evident in metropolitan locations [21,22], with specific challenges associated with caregiving in rural regions of Australia given its large land mass and sparse population. For example, New South Wales (NSW) is the most populous state in Australia with around 7.1 m people [23]. However, this population is spread across a geographic land mass of 800,000 square kilometres [24]—approximately twice the size of California and three times the size of the United Kingdom—and while there are 1.3 m people resident outside of the greater metropolitan regions [25] the population density is very low. The geographic spread of the population means that there are considerable distances between rural towns, and it can result in significant problems associated with accessing services and support infrastructures for both carers and the people they support [26]. Transport is very difficult, particularly for those in rural areas who need to travel for employment and services [18,27], and is an additional barrier to accessing social supports [28]. Individuals and their carers in rural areas often have to wait long periods of time for services or travel considerable distances to access them [29]. This poor availability and associated delays can then compound medical problems, due to complications that have arisen as a result of postponement of treatment and support [30,31,32], which in turn can increase the burden on caregivers.

### 1.2. Objectives

While caregiving is known to be commonplace across Australia, there remains limited knowledge of rurally-based carers, the challenges they face in carrying out the caregiving role, and the wider impact the provision of care has on the carer’s personal life. Using a purpose-developed survey tool, this study specifically explores how the provision of care in rural settings may influence employment and social support opportunities, as these two factors can be more acute given the lack of opportunities in rural regions compared to metropolitan areas of Australia.

## 2. Methods

### 2.1. Study Design

A cross-sectional survey was designed with demographic and socioeconomic measures used routinely by the Australian Bureau of Statistics (ABS) and the Australian Institute of Health & Welfare (AIHW). These questions specified the respondents’ age, gender, employment, caregiving status, condition of and relationship to the care recipient, postcode, residency status, and distance and frequency travelled to provide care. Additional purpose-developed questions were included that examined access to and participation in carer support groups. Open-ended comments sections were provided to allow participants to describe any issues and problems associated with caregiving including employment, travel, residency, carer support groups and any other general information. A pilot was conducted of both the print and online formats, with minor modifications being made to clarify some questions based on feedback from the pilot.

### 2.2. Participants

The inclusion criteria for the project was any individual providing care for someone with a long-term health condition or disability living in regional or rural regions of northern New South Wales (NSW). Caregivers for individuals who were experiencing ageing-related issues were included, but were categorised by the specific health-care condition (i.e., physical, cognitive, etc.) rather than under a generic label of “ageing issues”. Formal ethical approval was provided by the University of New England’s Human Research Ethics Committee (HREC Approval No.: HE13/130).

### 2.3. Setting

Rural and regional areas were defined in accordance with the Australian Geographical Classification System of major cities, inner regional, outer regional, remote, and very remote areas [33]. Northern NSW was defined as all areas situated north of Newcastle, which is located at approximately the mid-point of the state’s coastline and is the northern-most metropolitan area. The rationale for the selection of northern NSW was a lack of research studies over this very large geographical area which includes both regional and rural settings with limited services. A secondary consideration was the ongoing large-scale study around Newcastle and surrounds [34,35] which also covers regions south to Sydney (Australia’s most populous city and the capital of NSW).

### 2.4. Recruitment

To overcome the challenge associated with the wide geographical dispersal of carers in northern NSW, a community-based approach was used for recruitment. A combination of techniques including social media, posters in community facilities including clinics of local doctors, a combination of electronic and traditional media sources (radio, local newspapers, community newsletters of local agencies) were used to both promote and provide information on the study. Participants were given a choice of either responding online or they could opt to complete a print version of the questionnaire, which was provided along with a postage-paid return envelope. An incentive of a $5 grocery voucher was offered to participants. To ensure respondents anonymity, all hard copy surveys had a tear-off back page for address details which was posted back in a separate reply-paid envelope. The online survey was structured to direct participants to a detached online collector where address details were recorded separately to the survey data.

### 2.5. Analyses

Prior to detailed analyses of the data set, verification of data integrity was undertaken by performing consistency checks using SPSS v23 (IBM Corporation, Armonk, NY, USA). The level of rurality for the sample was determined using the Accessibility Remoteness Index Australia (ARIA) model [36]. An algorithm that matched postcode information against the ARIA classification was used to create four categories—inner regional, outer regional, remote and very remote. Health conditions were an open response question in the survey and were subsequently condensed into seven categories: physical health; mental health; cognitive impairment; physical and cognitive; physical and mental; cognitive and mental; and physical, cognitive and mental health.

Data from the open-ended response categories supplied as comments by the respondents were thematically coded by a team of three researchers using methods in line with the recommendations by Boyatzis [37]. Coding was undertaken separately and then compared, with any disagreements determined through discussion. To provide context to these qualitative responses, but to also ensure confidentiality of participants, each quoted statement has generic identifiers of gender, age, type of care provided and geographic location. Any situation in which a person’s name or other identifiable feature, such as a town or service name, was mentioned in a quote, it has been replaced with a string of asterisks (********).

## 3. Results

A total of 242 participants completed the survey. A postcode check indicated that 17 respondents from major NSW cities had completed the online survey. These 17 ineligible responses were removed yielding a final analysis sample of 225 respondents.

### 3.1. Carer Profile

The mean age of the 225 participants was 52.7 years (SD 14.4), with a range from 21 to 86 years. The gender distribution for study participants was predominantly females (*n* = 191), who comprised 86% of the sample (see Table 1). The majority of the sample (67%, *n* = 146) was resident in the outer regional areas as classified by ARIA, followed by 18% (*n* = 39) from inner regional areas, 6% from remote (*n* = 14) and 9% (*n* = 20) from very remote areas (see Table 1 below). Due to the small numbers of participants, data on remote and very remote categories are pooled together. Examples of geographic locations of participants included Broken Hill (Far Western NSW, approximate population—18,000), Moobi (Upper Hunter region, population—250), Byron Bay (North Coast, Population—5000), Armidale (Central Northern NSW, population—21,000) and South West Rocks (Mid-North Coast, population—4500).

The majority of the respondents reported their carer status as primary carer (83%, *n* = 179), with only 9% identifying themselves as secondary carers. Of the remaining 8%, most participants (6%) provided multiple responses, with 2% characterising themselves as “other carer”. The most common relationship was a parent caring for a child (43.2%), followed by spousal/partner care (36.2%) and children caring for parents (12.7%), with 8% reporting as other relationships (see Table 1). A small minority of participants (8%) indicated that they were responsible for providing informal care to more than one person. Being the primary carer for more than one family member with different health conditions added to the complexity of the situation and was noted as increasing the inability for the carer to cope with daily events. One carer outlined the problems he faced by noting: “*Due to my wife’s disability it is quite hard to know what the next day will bring and with my grandson also suffering mental health issues it adds to the pressure*” (Male, aged 58, Caring for Partner and Caring for Grandson, Outer Regional).

The health conditions for which care was provided were collated into seven categories. Physical health conditions were the largest portion of the sample at 37.7%, while cognitive conditions comprised 22.3%, mental health issues 12.6% and 27.4% were combinations of the above categories. The largest of the reported co-morbidities was physical and cognitive conditions (18.6%), followed by physical and mental health (6%), cognitive and mental health combined (1.9%) and a physical, cognitive and mental health (0.9%). Examples of reported multiple conditions included “*dementia + prostate cancer + recurring chest infections*” and “*Down syndrome, Autism Spectrum Disorder, Mild/moderative conductive hearing loss, Vision*”.

### 3.2. Employment and Caregiving

The age profile of the participants indicated that a majority would be considered to be “mid-career” in employment terms; however less than half (45%, *n* = 98) of the sample reported being employed (see Table 2 below). This contrasts with the August 2018 unemployment rate in NSW of 4.7% [38].

The response rate for employment type was higher than the response rate for whether the respondent was employed, perhaps indicating either a recent resignation or upcoming return to the workforce. Approximately one-third (29.2%) of the respondents indicated that they were in permanent employment, 17.4% were casually employed, 4.9% on contract work, 9% were self-employed, and 20.1% indicated they were employed in more than one of the above categories. There were 28 participants (12%) who provided additional information as they felt that their work situation did not fit any of the above employment categories. These comments primarily either reflected a desire to work, but an inability to do so due to their carer requirements, or the belief that their employment was as a full-time (but unpaid) carer and that it should be recognised as such.

Many respondents commented on problems associated with attempting to combine employment with the somewhat unpredictable nature of caregiving. Exemplar quotes that indicate the underlying concerns about employment and caregiving are included in Table 3 below.

Even the carers who reported part-time work options indicated that paid employment remained difficult, especially in situations where the family member had multiple serious health issues (see quote #2). In other instances, the nature of self-employment as a small business operator became unviable due to inability to work throughout the week. Even after the caring role had finished, individuals still reported ongoing problems in relation to employment. Some carers reported that they found it difficult to get back into the employment market after years of caring (e.g., quote #4).

Although specific questions around personal finances were not asked in the questionnaire, pressure on the individual’s financial situation were brought up independently by respondents. One participant stated that: “*Financial stress has been impacting on me during the past week. My partner has not worked at all for about five years but he does like to spend quite freely, so I always have to watch our finances carefully*” (Female aged 56, Caring for Partner, Outer Regional). In particular, both the inadequacy and difficulty in obtaining government payments to cover the needs and wants of the participants was reported: “*I also found it very difficult to get onto carers payment. The extra stress that created was not welcome*” (Female, aged 50, Caring for Child, Inner Regional).

The impact of providing care on their own health was also noted by participants. There were reports of physical health issues manifesting in problems, such as headache and lack of sleep, which naturally impact on the individual’s ability to work effectively and efficiently: “*I am currently exhausted and just can’t catch up. I care for people every day at work and then again at home and losing hours where I just can’t wake up or fall asleep or suffer migraines*” (Female, aged 36, Caring for Child, Inner Regional). The emergence of mental health issues associated with the burden of caring were also identified, along with associated dysfunctional coping behaviours, such as using alcohol or drugs. One participant described their situation and their ongoing problems as follows:

But others do not “understand” the emotional and physical toll it has taken on me. I am now trying to get my life back but find it extremely difficult often drinking alcohol to dull the pain and anxiety suffering from rashes that appear to have no cause other than “anxiety” according to doctors, back pain and worry as to where to now?

(Female, aged 40, Caring for Partner, Outer Regional)

The majority of the carers (75.5%, *n* = 142) were co-resident with the care-recipient. Of the remaining participants, close to half (*n* = 23) lived within a distance of 1–10 kms (approximately 6 miles), with a further 9 living within 10–20 kms. Only 3.7% of the total sample lived greater than 20 kms (approximately 12 miles) from the care-recipient. Around 20% of respondents stated that they needed to travel to specifically complete their caregiving responsibilities. Daily travel was a factor for 9.9% of the cohort, with multiple trips per week required by another 9.4% of participants. Less frequent travel was reported by remaining respondents, with 1.6% travelling on weekends only and 2.6% travelling once per month. However, these figures related to the regular travel that carers did to undertake their caring role and do not adequately take into account the additional travel that is then required to support individuals to attend non-consistent activities, such as medical appointments or other health-care support. That aspect of travel needs to be specifically examined in future research with rural caregivers. Table 4 (as below) outlines some of the key comments associated with challenges that travel presents for care provision.

### 3.3. Access to Carer Support Group

Respondents were asked about their knowledge of and involvement in local carer support groups in order to establish whether there were community-based support structures in place. Although a large proportion of carers (69.4%) indicated they were aware of support groups in their area, only 61 of the 225 participants (27%) indicated having membership of any support group (see Table 2). The free-text responses indicated that time constraints were one of the main impediments to active participation in support groups. One carer noted that “*When you provide 24 hr. care, the respite time you have is devoted to required shopping, appointments, etc.*” (Female, aged 63, Caring for Partner, Outer Regional). Other carers also identified time as the main problem:

*Not enough time. I have 4 children, a husband who works shift work and I work two days a week myself. My children are involved in local sports, art classes, etc. There is simply not enough time to attend support groups as well*.

(Female, aged 42, Caring for Child, Inner Regional)

*Time. I don’t have the time to join a support group. I basically cannot leave the house except for a three-hour period on Mondays when the housekeeper comes*.

(Female, aged 62, Caring for a parent and caring for a spouse, Outer Regional)

However, for some respondents there were other concerns regarding joining carer support groups that may be particular to rural settings. For some carers, particularly younger ones, the reasons went beyond time constraints to negativity expressed by care-recipients about their information being shared at carer support groups.

Over 30% of respondents indicated that they were unaware of any carer groups in their area. Many smaller communities do not have any carer support groups, and may also have limited opportunities for socialising beyond the local pub or club which can act as the focal community meeting point. Some carers did not see themselves fitting into this local lifestyle, or had financial restrictions that further increased their social isolation. Other participants, especially those single carers and carers of children with disabilities, expressed feelings of isolation as they had limited time to nurture social relationships.

Exemplar comments of these issues of loss of anonymity or social isolation are included in Table 5 below.

## 4. Discussion

### 4.1. Key Results

It is recognised that caregiving is demanding, both physically and emotionally, and can have cascading negative health effects when combined with reduced social interactions and income [39]. These issues sometimes do not stop until either the care recipient or the caregiver dies; the second situation often resulting in institutionalisation of the care recipient [39,40]. The impact of caregiving on physical and mental health of carers has been described in greater detail in an earlier publication [41]. In this paper, the focus is on other important facets of caregiving; its effect on employment and carers access to social support structures.

The results indicated that the majority of carers were aware of the social supports available in their local rural community, but did not access them, leaving the carers vulnerable to marginalisation. Access to support services, such as carer groups, were also rarely accessed due to a mix of factors including inaccessibility, poor timing and a lack of anonymity. The participants also identified specific barriers to the provision of care in rural areas, including the significant impact associated with travel requirements to assist with care, and the impacts of care provision on the respondents’ own personal health. Financially, there was considerable evidence of hardship, and significant employment consequences associated with inflexibility and limited job options in rural locations. Problems associated with employment were noted as resulting in financial pressures and associated personal stress and anxiety for the caregivers.

Many of the current findings generally align with reports based on SDAC surveys by Australian Bureau of Statistics and other agencies. However, while the identified issues are not necessarily limited to rural areas, it would appear that the lack of opportunities and inflexibility evident in rural areas exacerbates these problems for non-metropolitan residents. This study specifically notes the issues facing rural residents, which have not necessarily been accurately captured in previous research.

### 4.2. Unemployment and Under-Employment

One of the main issues raised by participants in the comments section was unemployment and/or underemployment. Carers of working age, who comprised the majority of the sample, indicated that they felt disadvantaged when it came to participation in the labour force and career progression. It is known that unemployment is linked to poor health outcomes [42], that opportunities for employment in rural areas are limited compared to metropolitan locations [43] and that the need to provide care can further exacerbate these existing disadvantages [44].

In the Australian context, research has shown that female carers tend to have greater disadvantage in terms of employment, with many women tending to opt for leaving the workforce altogether or changing jobs to part-time or casual positions [45,46]. The health conditions for which care is provided can be unpredictable, leading to frequent absence or unpaid leave. The caring duties at home may lead to a lack of performance, which can in turn see caregivers either having to resign or face formal disciplinary action [47,48]. This combination of factors continues throughout the life of rurally-based primary carers; they have poor personal health outcomes and reduced income-earning opportunities as they have to provide care, but they also then miss out on the compulsory employer contribution to superannuation. This situation is not ideal for either the individual or government, as the carer is then often completely dependent on the aged care pension when they reach retirement age [49].

The lack of employment opportunities can also result in financial pressures and associated stress, and this issue was raised by several respondents. The inability for many carers to work due to their responsibilities, as well as the difficulties in receiving access to government support, was a very noticeable feature in the open-ended comments section of the survey form. Carers’ financial disadvantage is not a new concept [50]. However, not much has changed in the past decade and can be argued that the model in Australia is set up in such a way that financial marginalisation is inevitable. In 2016, the Government carers payment was approximately AUD $21,000 per annum (pa) for a single carer, and AUD $31,000 pa for a couple. However, there are many restrictions on access to this payment with limits on assets, income and health conditions which must meet rigid criteria [51]. These figures also show that a person providing full-time care and reliant on the Carers Payment would fall under the national poverty line of $22,000 as a single or $46,500 as a couple [52]. These financial problems result in situations where carers have difficulty in paying for even basic services, such as fuel and phone bills, when compared to the mainstream population [18].

### 4.3. Carer Support

The stress associated with caregiving can sometimes be mitigated through the use of social support, including carer support groups, which may lead to better health and employment outcomes. Stoltz et al. [53] noted that many family carers like to network with peers and with groups of people in similar situations in order to reduce their social isolation, and to learn or share knowledge and experiences. Although many participants in the current study expressed knowledge of local carer support groups, very few reported that they were able to attend sessions. One reason cited for a lack of attendance at support groups was time. The role of caring impacted upon the participants’ capacity to access the very services designed to support their caring. The issue of time also related to the need to travel to access even basic services [18,54] and particularly specialist services [55]. A recent Australian study using phenomenological approach to issues in caregiving highlight three important challenges as enunciated by the carers as being time, worry and cost [56]. However, the lack of anonymity in small rural communities and concerns about causing further distress to the care-recipient were also major factors for individuals not accessing support services. This can then result in isolation or being disengaged from social activities out of the home [57]. Access to information and communication technologies has been found to increase quality of life of carers by saving travel time and expenditure [58,59] and another option may therefore be to consider the greater use of online technology in rural areas to enable participation from home without having to travel long distances or worry about lack of anonymity.

### 4.4. Limitations

It is acknowledged that the study sample was relatively small and caution is advised in generalising the findings across the diverse population of rural carers, and particularly in localities where support service infrastructure may vary to that of NSW and Australia. However, a recent study based on a large sample from the Netherlands found that responder bias is less likely to be an issue in studies of caregiving as carers who have a significant caregiving responsibility are represented adequately in survey research, which is perhaps a reflection of trying to draw attention to the sometimes dire needs of caregivers [60]. In the current study, there is a strong bias towards female respondents of middle-age, and while this does represent the carer demographic in Australia, it would need to be considered when comparing these results to cohorts of carers elsewhere in the world. Nonetheless, it is important to acknowledge that most large-scale studies do continue to find a similar gendered-dimension to informal family caregiving.

### 4.5. Recommendations

As noted in the report commissioned by Carers Australia [61], further gains in life expectancy, as well as increased likelihood of additional health problems especially towards the later part of life, will further stretch demand for informal caregiving. Therefore, it is essential that carers be better supported to maintain their employment options and health, and there are several key recommendations arising from this research regarding future projects and more targeted support for rural care-givers.

#### 4.5.1. Future Research

The issues of geographical distance and social isolation are key factors that need to be accounted for when developing models of support to better assist rural carers to successfully balance their employment and caregiving roles. While these issues may also be experienced by some metropolitan carers, the limited resources in small towns present additional barriers for rural carers. It is recommended that future research further specifically explore the problems associated with employment, travel and support groups for rural carers. This could include quantitative research that compares the relative impact of each of these factors, or qualitative in-depth personal interviews to unpack the initial findings from the open-ended responses and gain a more thorough understanding of rural carers’ challenges.

#### 4.5.2. Support for Caregivers

The greater availability of and more flexibility in accessing a variety of support groups at suitable times is a clear need for rural carers. It is recommended that community-based services in these areas re-conceptualise their structures to allow flexible delivery models in order to better meet the needs of this group. In particular, the previous literature has not satisfactorily identified the concerns of rural carers regarding anonymity in small communities, and the impact this concern has on individuals desire to access carer support groups (when available). The lack of flexible work opportunities was very evident for rural carers, and a recommendation is for further government supports to be made available in order both to alleviate severe economic marginalisation and as a proactive preventative measure for the rural carer cohort.

It is known that many care-givers, both rural and metropolitan, have to travel to provide daily assistance. However, what is not adequately understood is impact of the additional travel needs to support individuals to attend non-consistent activities, such as medical appointments or other health-care access support, in locations external to the home location. Additional government assistance to support travel, either through financial reimbursement or community-based transport solutions, would appear to be beneficial; however it is premature to make specific recommendations until the exact needs of this cohort are better understood.

## Figures and Tables

**Table 1:** Profile of rural carers.

Demographic Variables	*n*	%
Age (mean, SD)	52.7 (14.4)	---
Sex		
Males	31	14
Females	191	86
Location		
Inner Regional	39	17.8
Outer Regional	146	66.7
Remote/Very Remote	34	15.5
Caregiving Status		
Primary	180	82.9
Secondary	19	8.8
Other	5	2.3
Multiple Response	13	6.0
Supported Health Conditions		
Cognitive	48	22.3
Mental Health	27	12.6
Physical	81	33.7
Physical/Cognitive	40	18.6
Physical/Mental Health	13	6
Cognitive/Mental Health	4	1.9
Physical/Cognitive/Mental Health	2	0.9

**Table 2:** Caregiving profile of study respondents.

Caregiving Profile	*n*	%
Work Status		
Yes	98	45.2
No	119	54.8
Employment Type *		
Casual	25	17.4
Permanent	42	29.2
Contract	7	4.9
Self Employed	13	9.0
Multiple Types	29	20.1
Other (participant nominated)	28	19.4
Residence		
Co-Resident	142	75.5
Non-Resident	46	24.5
Distance Travelled		
1–10	23	50.0
11–20	9	19.6
>20	7	15.2
Multiple Answers	7	15.2
Frequency of Travel		
I don’t travel	138	72.3
Daily	19	9.9
Multiple times per week	18	9.4
Weekends only	3	1.6
Once a fortnight	3	1.6
Once a month	5	2.6
Multiple Answers	5	2.6
Aware of Support Groups		
Yes	152	69.4
No	67	30.6
Member of Support Groups		
Yes	61	34.5
No	116	65.5

* More people responded to employment type than those who reported they worked. This variation may be due to people not working at the time, but who were planning on returning to their previous profession of choice.

**Table 3:** Respondent commentary on employment and care-giving.

#	Respondent Demographics	Comments
1	Female Aged 53 Caring for Child Inner Regional	*Need the flexibility to not work when my daughter is unwell or has appointments; or when I am unwell!*
2	Female Aged 52 Caring for Partner Outer Regional	*I was working as an Enrolled Nurse at the local hospital but resigned to care for my husband aged 51 years, who has had Type I diabetes. He also has IHD (ischemic heart disease) which has resulted in heart bypass surgery x4 grafts, and x5 stents. He also has osteoarthritis, Raynaud’s Syndrome, autoimmune facial dermatitis, visual impairment, industrial deafness, and also has mental health issues, including PTSD, and currently awaiting formal diagnosis of bipolar.*
3	Female Aged 46 Caring for Child Outer Regional	*Business no longer viable largely due to caring for two people for years.*
4	Female Aged 40 Caring for Grandparent Outer Regional	*I put my life on hold to care for my grandmother for 3 years. She has now passed away. I feel let down! I am finding it hard to get employment again.*

**Table 4:** Respondent commentary on travelling distance and care provision.

#	Respondent Demographics	Comments
5	Female Aged 52 Caring for Partner Outer Regional	*I regularly do all the driving to medical appointments, and also am responsible for the prescriptions. Travel is by private car, up to 600 kms (round trip, if we are seeing cardiologist), 300 kms (round trip to see endocrinologist), and 80 kms (round trip) to see a GP.*
6	Female Aged 25 Caring for Parent Outer Regional	*I provide assistance in the care of my father, who lives approx. 800 km away. My mother is the primary carer, but I am regularly called upon regarding appointments, interpreting diagnoses, and providing mental support for my father.*
7	Female Aged 50 Caring for Parent Outer Regional	*I am primary carer for my father since his colorectal surgery (due to cancer) in 2009. However, in the past few years my mum has been unwell and I also assist her and dad (they live together) with everything in their day to day running of their home, personal lives and looking after their animals. When I am not working at my casual employment 2 days a week I am at mum and dad’s home probably 90–95% of the time helping them. Even though it is only approximately 5 km (5 mins) from my home to theirs, sometimes it is just easier for me to stay at their home if one or both of them are unwell. My sister is also a wonderful help however she travels 50 km each way to work (full-time) and works extremely long hours.*
8	Female Aged 66 Caring for Parent Outer Regional	I have been a carer for the past 15 years. Initially I visited both parents in ******** for 4 days once a month which was a time and financial burden. Six years ago, my parents came to live in ********. My father had dementia and was living in a dementia-specific unit. I visited him daily until he died in 2010. My mother lived with my husband and I for 4 years after she had bowel surgery for cancer. In 2012, she moved into low care accommodation when she wasn't safe to leave alone (angina, falls). I visit her every day.

**Table 5:** Respondent commentary on anonymity and social isolation with respect to accessing Carer Support Groups.

#	Respondent Demographics	Comments
9	Female Aged 68 Caring for Partner Outer Regional	*I feel I cannot discuss private issues, such as reasons for feeling miserable with people who know me in this small town. There is lack of anonymity. Friends provide me with relaxation and a coffee break.*
10	Female Aged 38 Caring for Partner Outer Regional	*I don't have time, the concept is not supported by my partner who sees the sharing of any personal information as intrusive and risking interference by other people.*
11	Female Aged 44 Caring for Partner Remote/Very Remote	*Isolation and no children make it hard to mix especially when the carer is a non-drinker or non-smoker and we are not flush with money to waste of stuff and unnecessary meals outings etc.*
12	Female Aged 44 Caring for Child Outer Regional	*I know that I do a great job as a single mother and have little problems with self believe. I feel completely overwhelmed raising 2 children with disabilities. I struggle financially, physically and emotionally. I am socially isolated. I struggle with maintaining friendships and relationships. I put in 120% and get about 20% back.*
